# Circulating mtDNA and Impaired Intestinal Barrier after Gastrointestinal Surgery Are Correlated with Postoperative SIRS

**DOI:** 10.3390/genes13111933

**Published:** 2022-10-24

**Authors:** Can Kong, Wei Song, Jun Ren, Dingshan Zhou, Jiazheng Li, Renshen Xiang, Tao Fu

**Affiliations:** 1 Department of Gastrointestinal Surgery II, Renmin Hospital of Wuhan University, Wuhan 430060, China; 2 Intravenous Drug Dispensing Center, Renmin Hospital of Wuhan University, Wuhan 430060, China; 3 Department of General Surgery, Qingdao Municipal Hospital, Qingdao 266071, China

**Keywords:** mtDNA, DAO, SIRS, intestinal barrier dysfunction

## Abstract

Background: This prospective study aimed to explore the correlation between circulating mitochondrial DNA (mtDNA), intestinal barrier function impairment, and postoperative SIRS in patients undergoing gastrointestinal surgery. Methods: Patients were recruited into this study after signing an informed consent form. Circulating mitochondrial DNA and serum DAO concentrations were measured preoperatively and on day 1 and day 7 postoperatively. Postoperative vitals, routine tests, and biochemical indicators were recorded in detail. Results: Forty patients undergoing gastrointestinal surgery were recruited for and completed this study. Patients were divided into non-fever, fever, and SIRS groups according to their postoperative temperature and other corresponding indexes. The mtDNA was expressed as the number of PCR cycles using three specific sequences. Circulating mtDNA tended to increase in patients after gastrointestinal surgery, but the difference was not significant. Nevertheless, mtDNA in the SIRS group was significantly higher than in patients in the fever and non-fever groups (*p* < 0.05). Serum DAO showed a trend of increase on the first day after surgery compared with that before surgery, but the difference was not significant (*p* > 0.05). However, patients in the SIRS group showed a significant increase (*p* < 0.05) compared with the others. Both circulating mtDNA and DAO showed a downward trend on the seventh day after surgery. Conclusions: Circulating mtDNA presented a trend of increase after gastrointestinal surgery, and the degree of the increased fold was related to the extent of the inflammation response. In general, the intestinal barrier damage indicator DAO was the same as mtDNA and tended to increase after gastrointestinal surgery and then gradually decrease, which may play a synergistic role in inducing postoperative fever and SIRS.

## 1. Introduction

Mitochondria are the key organelles of cellular energy metabolism. At the same time, mitochondria determine cells’ physiological function and serve a vital role in the immune response [1]. Important damage-associated molecular patterns (DAMPs), such as mitochondrial DNA released after mitochondrial damage caused by severe trauma or abdominal surgery, may impact the respiratory chain, enhance oxidative stress, and activate systemic inflammatory responses through diverse inflammatory signaling pathways [2,3,4]. Severe cases may even lead to SIRS (systemic inflammatory response syndrome), MODS (multiple organ dysfunction syndrome), and death [5]. By testing the postoperative circulating mtDNA levels in patients following pancreaticoduodenectomy, Pencovich et al. [6] found that circulating mtDNA surge is correlated with patients’ postoperative inflammatory response, suggesting that abdominal surgery may contribute to patients’ postoperative inflammatory response by releasing mtDNA. One of our recent reviews also detailed the pathophysiological mechanisms of the inflammatory response induced by mitochondrial damage [7].

Intestinal homeostasis involves the interaction between the intestinal flora, the protective mucus layer, and various intestinal cells (intestinal epithelial cells, myeloid cells, and lymphocytes) [8,9]. The crucial role of intestinal homeostasis is particularly important due to the large direct contact of the intestinal lumen with the “external” environment. When this balance is disrupted, the possibility of endogenous danger signals increases. Those signals are generated by microorganisms and their metabolites, as well as abiotic, xenobiotic, and self-interactions in the intestinal lumen [10]. 

It has already been found in animal experiments and clinical studies that even minor intestinal manipulation can cause increased permeability of the intestinal mucosa. Similar phenomena have been observed in open cholecystectomy, laparoscopic cholecystectomy, and even in cardiac surgery, chronic liver cirrhosis, severe trauma, and other critical illness [11,12,13,14,15,16]. As a result of increased permeability, intestinal bacteria, endotoxins, and other harmful substances migrate into the bloodstream and trigger a series of pathophysiological processes, including SIRS and MODS.

Diamine oxidase (DAO) is only synthesized in the epithelial cells of the intestinal villi. It breaks down polyamines such as histamine and controls the proliferation of the mucosa [17]. The release of DAO from damaged mucosal cells increases their serum concentration [18]. Therefore, this indicator has a certain value in assessing the degree of intestinal barrier damage. Both our research and other, previous studies have confirmed the value of DAO in assessing intestinal barrier function impairment after gastrointestinal surgery [19,20,21].

In this study, we aim to assess the relationship between mtDNA, impaired intestinal barrier, and SIRS in patients undergoing gastrointestinal surgery so as to explore its clinical application to forewarn SIRS and intestinal barrier dysfunction after gastrointestinal surgery.

## 2. Patients and Methods

### 2.1. Ethics Statement

Patients undergoing gastrointestinal surgery were recruited for this study, and this study protocol was approved; all patients signed an informed consent form prior to performing any study-related procedures.

The inclusion criteria were: patients were diagnosed with colorectal cancer, gastric cancer, or gastrointestinal disorders (gastrointestinal mesenchymal tumor, strangulated intestinal obstruction, intestinal torsion, Crohn’s disease, etc.) needing surgical treatment; age from 18 to 90 years; hepatic function Child-Pugh classification of grade A or B; and clear consciousness and ability to communicate with researchers in normal language.

The exclusion criteria were: patients who were not undergoing surgical treatment; significant abnormalities in preoperative biochemical tests: dyslipidemia (total triglycerides > 2.26 mmol/L or total cholesterol > 6.2 mmol/L) or renal impairment (creatinine > 122 µmol/L or urea > 10.7 mmol/L); those who had recently received radiotherapy; those who had recently received hormone therapy; presence of preoperative fever (temperature over 37.2); patients on anti-pyretic therapy (e.g., paracetamol, Tylenol, aspirin, etc.); and co-morbidities such as diabetes, autoimmune diseases, and inflammatory diseases.

The primary outcome was postoperative fever; secondary outcomes involved SIRS, MODS, and death. SIRS was defined according to the American College of Chest Physicians/Society of Critical Care Medicine [22]. MODS was defined according to the Denver Postinjury Multiple Organ Failure criteria with a score of 4 or higher [23].

### 2.2. Blood Sampling

Blood samples were collected on admission to the hospital, day 1 after surgery, and day 7 after surgery. These blood samples were collected into ethylenediaminetetraacetic acid (EDTA)-containing tubes and centrifuged twice at 3000 revolutions per minute (rpm), and 10,000 rpm, both for 10 min. The upper part of the plasma was transferred into another clear tube and stored at −80 °C before DNA extraction or enzyme linked immunosorbent assay (ELISA). 

### 2.3. Measurement of mtDNA in Plasma 

DNA was isolated from plasma using the QIAamp DNA Blood Midi Kit (Qiagen, Valencia, CA) according to the manufacturer’s protocol. Quantitative real-time polymerase chain reaction (qPCR) was then performed using Universal Blue qPCR SYBR Green Master Mix (Yeasen Biotechnology, Shanghai Co., Ltd. CN). The primers for qPCR analysis of mtDNA sequences were *Human cytochrome B* F: ATG ACC CCA ATA CGC AAA AT R: CGA AGT TTC ATC ATG CGG AG; *MT-ND2* F: CAC AGA AGC TGC CAT CAA GTA R: CCG GAG AGT ATA TTG TTG AAG AG; *COX1* F: TCA TCT GTA GGC TCA TTC R: GCG ATC CAT ATA GTC ACT. These mitochondrial genomic regions for primers of qPCR were described previously [4,24]. The total volume of the reaction system was 20 uL, and the relative plasma mtDNA abundance was denoted as the threshold cycle (Tc).

### 2.4. Assessment of Plasma DAO 

Human serum DAO concentration was measured using the ELISA Kit provided by Cusabio Biotech Co., Ltd. (Wuhan, China). We detected mtDNA and DAO in preoperative plasma as negative controls.

### 2.5. Statistical Analysis

Statistical analyses were performed using the SPSS 25.0 statistical software (SPSS Inc. Chicago, IL, USA). Figures and tables were performed by the GraphPad Prism 8.0 (GraphPad Software, Inc., San Diego, CA, USA) and Word 2016 software (Microsoft, Redmond, WA, USA), respectively. Analysis of variance (ANOVA) by Tukey’s test was used to analyze differences between groups. The data were expressed as mean ± SD. A *p*-value < 0.05 was considered statistically significant.

## 3. Results

During the study period, 40 consecutive patients were enrolled. Patients were divided into non-fever, fever, and SIRS groups according to their postoperative temperature and other corresponding indexes (heart rate > 90/min; respiratory rate > 20/min or PaCO_2_ < 32 mmHg; white blood cell count >12 × 10^9^/L, <4 × 10^9^/L, or >10% immature (band) forms). The characteristics of the patients are indicated in Table 1. There were no serious complications and no deaths, except for one patient who had bleeding on the wound around the anastomosis after surgery and underwent a second operation. The average ages of the groups were 57 ± 15.18, 62.52 ± 6.64, and 72.17 ± 4.79, respectively. There was no statistically significant difference between the non-fever group and the fever group, but there were significant differences between the non-fever and SIRS groups, as well as between the fever group and the SIRS group (*p* < 0.05).

### 3.1. mtDNA Levels

**Figure 1:**
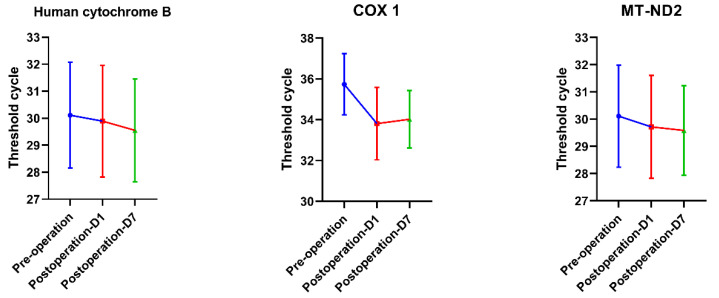
The results of mtDNA-PCR indicated that the Tc value on the first day after surgery was lower than that before surgery, and it showed an upward trend on the seventh day but was still lower than that before surgery. However, statistical analysis showed no significant difference (*p* > 0.05). Note: the lower the Tc value, the higher the mtDNA concentration.

The concentration of mtDNA in plasma is quantified as PCR Tc, and the amount of DNA doubles with each cycle until the detection threshold is reached. mtDNA in peripheral blood was measured before the operation, on the first day, and on the seventh day after the operation. Template DNA was considered to have a Tc value of 40 as the limit of detection. Therefore, all serum samples had Tc values below 40. The test results showed an upward trend in mtDNA levels on the first postoperative day in contrast to the preoperative day, and a downward trend on the seventh postoperative day compared to the first postoperative day, but none of these differences were significant (*p* > 0.05) (Figure 1).

### 3.2. mtDNA Levels in Three Groups

Patients were divided into three groups according to whether they had a fever or met the diagnostic criteria of SIRS after surgery, namely, the non-fever group (*n* = 9), fever group (*n* = 25), and SIRS group (*n* = 6). Statistical analysis showed that the mtDNA level in the SIRS group was significantly higher than in the no fever and fever group (*p* < 0.05) in three kinds of primers (Table 2).

### 3.3. DAO Concentration

**Figure 2:**
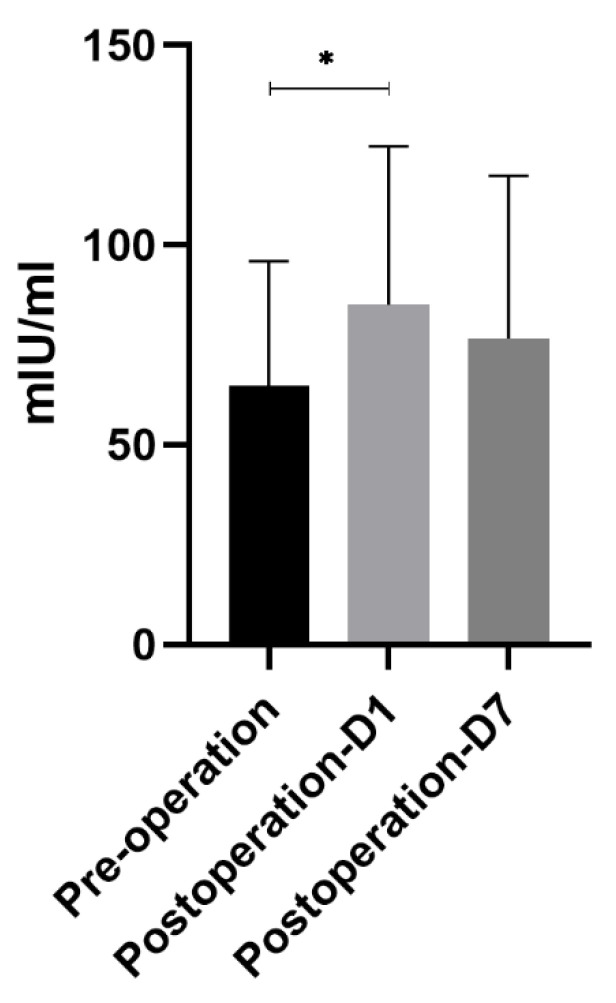
DAO concentration. Similar to the trend of mtDNA, DAO concentration also increased significantly on the first postoperative day compared with before surgery—64.65 ± 31.22 mIU/mL vs. 85.03 ± 39.61 mIU/mL (*p* < 0.05)—and gradually decreased to the preoperative level on the seventh day, reaching 76.54 ± 40.73 mIU/mL. * means *p* < 0.05.

Similar to the trend of mtDNA, DAO concentration also increased significantly on the first postoperative day compared with before surgery—64.65 ± 31.22 mIU/mL vs. 85.03 ± 39.61 mIU/mL (*p* < 0.05)—and it gradually decreased to near preoperative levels on the seventh postoperative day, reaching 76.54 ± 40.73 mIU/mL (Figure 2).

### 3.4. DAO Concentration in Three Groups

**Figure 3:**
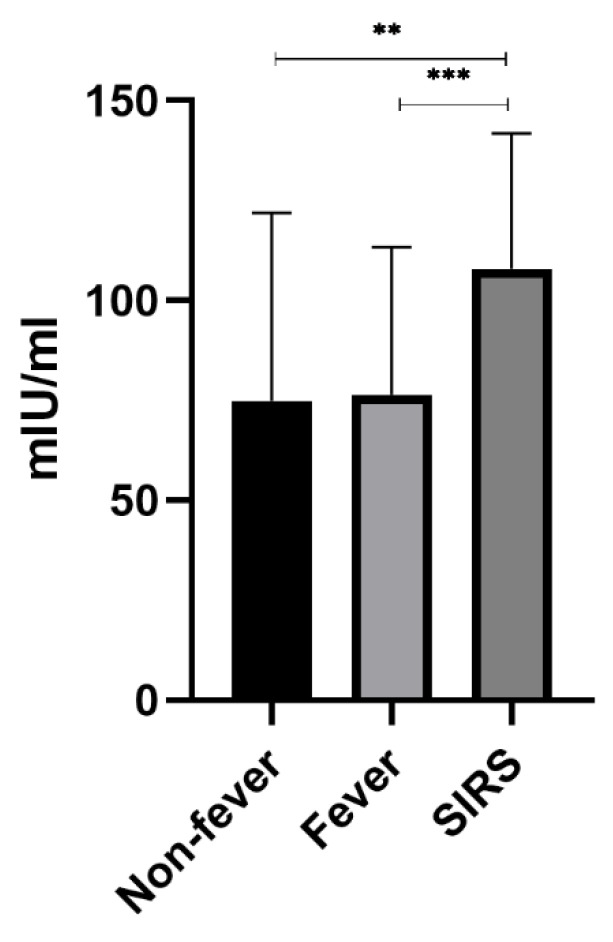
Association of DAO levels with fever or SIRS serum DAO level was detected, segregating patients based on the occurrence of fever and SIRS, with statistical difference between Non-fever and SIRS and between Fever and SIRS, but no statistical difference between Non-fever and Fever. ** means *p* < 0.01. *** means *p* < 0.001.

Analysis of DAO level differences among the three groups showed that there were significant differences between the non-fever group and the SIRS group and between the fever group and the SIRS group (*p* < 0.05), while there was no significant difference between the non-fever group and the fever group (*p* > 0.05) (Figure 3).

### 3.5. Correlations between mtDNA and DAO

Correlation analysis between mtDNA detected by three primers and DAO showed that there was no significant correlation between mtDNA and DAO concentration before operation; *human cytochrome B* and *MT-ND2* were significantly correlated with DAO concentration after operation. However, the correlation coefficient was minimal (Table 3). This indicates that postoperative mtDNA release and impaired intestinal barrier could be independent but synergistic factors in inducing postoperative fever or SIRS.

**Figure 4:**
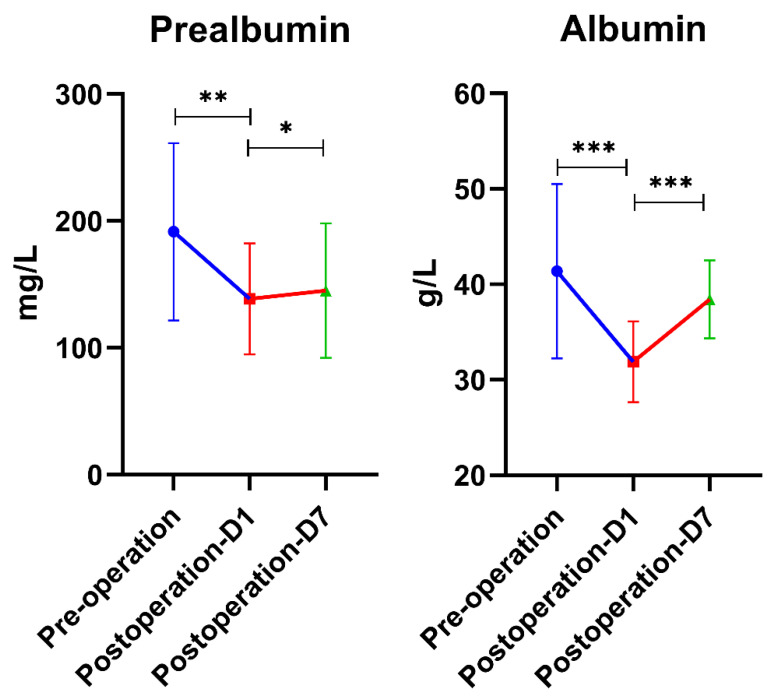
Serum prealbumin and albumin levels were detected; they were significantly decreased on the first postoperative day and gradually recovered on the seventh postoperative day but were still lower than the preoperative level (*p* > 0.05). * means *p* < 0.05; ** means *p* < 0.01; *** means *p* < 0.001.

In addition, we found that the concentration of albumin and prealbumin in patients decreased significantly on the first postoperative day compared with pre-operation, and we observed gradual regression to near preoperative levels by postoperative day 7 (Figure 4). While levels of postoperative albumin, which reflect short-term nutritional status, were lowest in the SIRS group, they were not significantly different from the other two groups (*p* > 0.05), suggesting that nutritional status may be related to the inflammatory response of surgical patients, but further verification is needed. 

## 4. Discussion

Previous studies have shown that fever and inflammation after trauma or surgery are frequent and greatly influence patient clinical outcomes [5,25]; development of inflammation is associated with poor prognosis, e.g., increased use of intensive care, longer hospital stays, and increased mortality. Nevertheless, inflammation remains a neglected and underreported pathophysiological change in patients with severe trauma or surgery, especially in older patients [24,26]. mtDNA, as an important damage-associated molecular pattern (DAMP), could enhance oxidative stress and initiate systemic inflammatory responses. In some severe cases, mtDNA can lead to sepsis, MODS, and even death [6,27,28]. 

The intestinal epithelium is a multicellular interface close to a complex microbial environment and completes renewal every 3–5 days [29]. The intestinal epithelium is richly and specifically supplied by a vascular system, but this high metabolism and the specific villi microvascular structure make the intestinal mucosa particularly sensitive to microenvironmental disturbances [30]. During inflammation, alterations in blood flow, vascular integrity, and capillary pathological permeability predispose to impaired tissue oxygenation. Thus, various causes of inadequate intestinal perfusion can lead to increased intestinal permeability and impaired intestinal mucosal barrier function [31,32].

In this study, we found that after gastrointestinal surgery, patients in the SIRS group had significantly higher levels of mtDNA and DAO than before surgery. mtDNA and DAO in patients with SIRS were significantly higher than before surgery, and patients with postoperative fever but not yet meeting the diagnostic criteria of SIRS also showed an increasing trend, suggesting that mtDNA may play an active role in the progression of postoperative fever and SIRS. The increase of DAO after surgery may reflect the impairment of intestinal barrier function, which may lead to the displacement of intestinal flora and induce SIRS or even MODS together with mtDNA. In addition, it was found that mtDNA and DAO had the same upward and downward trends. However, there was no significant correlation between them, suggesting that postoperative mtDNA release and impaired intestinal barrier might be independent but synergistic factors in inducing postoperative fever or SIRS. Nonetheless, this hypothesis merits further study. In addition, we found that the occurrence of postoperative fever and SIRS was positively correlated with patient age, i.e., the older the patients are, the more likely they are to develop postoperative fever and SIRS, further demonstrating that older patients are less tolerant of surgical trauma than younger patients.

Some studies have found increased inflammatory responses in patients with disease-related malnutrition [33], and our study observed a similar phenomenon. That is, both albumin and prealbumin showed a decreasing trend after surgery. The decreased degree of prealbumin in the SIRS group seems higher than that in the non-fever group and fever group, but the difference is not significant (*p* > 0.05), and further research is needed (Figure 4). Therefore, timely and effective nutritional support should be implemented for patients at risk of malnutrition after major gastrointestinal surgery. 

## 5. Conclusions

Postoperative fever and SIRS are associated with advanced age as well as hypoproteinemia. Circulating mtDNA tended to increase after gastrointestinal surgery, and the fold of the increase was related to the degree of inflammation response. DAO, an indicator of intestinal barrier damage, was the same as mtDNA, with a general trend of increase after gastrointestinal surgery and then a gradual decrease, which may play a synergistic role in inducing postoperative fever and SIRS. However, as this study is a small single-center study, its clinical application value needs further validation.

## Figures and Tables

**Table 1:** Individual Patient Characteristics.

Basic Information	Total (*n* = 40)	No Fever (*n* = 9)	Fever (*n* = 25)	SIRS (*n* = 6)	*p* Value
Age (years)	62.73 ± 9.93	57 ± 15.18	62.52 ± 6.6 *	72.17 ± 4.79 **	**0.01**
Sex ratio (male:female)	23/40	5/4	14/11	4/2	0.68
Types of surgery					0.77
Radical resection for gastric cancer	20	6	11	3	
Radical resection for colon cancer	10	1	7	2	
Radical resection for rectum cancer	8	1	7	0	
Enterectomy and anastomosis	2	1	0	1	

Legend: Values are expressed as mean ± SD. Statistically significant differences are shown in bold. *p*-values were calculated by the Mann–Whitney U test and the chi-square test. * means compared with non-fever group, *p* < 0.05. ** means compared with non-fever group, *p* < 0.01.

**Table 2:** Association of mtDNA levels with fever or SIRS.

mtDNA Levels	Non-Fever	Fever	SIRS
*Human cytochrome B*	29.60 ± 1.98	29.94 ± 1.95	26.62 ± 1.35 ** ^##^
*COX1*	33.74 ± 0.88	34.19 ± 1.71	30.75 ± 1.60 ** ^##^
*MT-ND2*	29.39 ± 1.92	29.84 ± 1.71	26.99 ± 1.20 ** ^##^

Legend: mtDNA = mitochondrial deoxyribonucleic acid; SIRS = systemic inflammatory response syndrome. Serum mtDNA levels for three different sequences were detected, segregating patients based on the occurrence of fever and SIRS, with statistical differences in every sequence. There were no significant differences between the non-fever group and the fever group (*p >* 0.05). Between the non-fever group and the SIRS group, and between the fever group and the SIRS group, there was a statistical difference in every sequence (*p <* 0.05). Each polymerase chain reaction cycle represents a doubling of the DNA quantity. ** means compared with the non-fever group, *p* < 0.01. ^##^ means compared with the fever group, *p* < 0.01.

**Table 3:** Correlations between mtDNA and DAO.

DAO	Preoperatively		Postoperatively	
	Correlation Coefficient	*p* Value	Correlation Coefficient	*p*-Value
*Human cytochrome B*	0.020	0.905	−0.159	0.037
*COX1*	−0.009	0.966	−0.282	0.075
*MT-ND2*	−0.108	0.514	−0.206	0.007

Legend: DAO, diamine oxidase. Plasma mtDNA level was quantified as PCR Tc; plasma DAO concentration was quantified as mIU/mL.

## Data Availability

The data that support the findings of this study are available from the corresponding author T.F., upon reasonable request.
